# A VAT1-related gene signature predicts radioresistance in gliomas

**DOI:** 10.1016/j.heliyon.2025.e42583

**Published:** 2025-02-08

**Authors:** Xia Shan, Zhiyan Sun, Ruoyu Huang, Kuanyu Wang, Xiaoguang Qiu, Pei Yang

**Affiliations:** a Department of Radiotherapy, Beijing Tiantan Hospital, Capital Medical University, Beijing, China; b Department of Neurosurgery, Beijing Tiantan Hospital, Capital Medical University, Beijing, China; c Department of Molecular Neuropathology, Beijing Neurosurgical Institute, Capital Medical University, Beijing, China; d Department of Gamma Knife, Beijing Tiantan Hospital, Capital Medical University, Beijing, China

**Keywords:** Glioma, Radioresistance, Immunosuppression, Prognosis

## Abstract

**Background:**

Radiotherapy is a vital postoperative adjuvant treatment for gliomas. However, radioresistance seriously affect the treatment efficacy. Excavating the feature of radioresisrtance in gliomas comprehensively are necessary.

**Methods:**

In the training set, 191 patients from the Chinese Glioma Genome Atlas (CGGA) were included, of which all patients had received postoperative radiotherapy. The epidemiological data and RNA sequencing data of 430 patients with whole grade glioma were obtained from the Cancer Genome Atlas (TCGA), which was used for validation.

**Results:**

Based on the Lasso regression analysis, five-gene signature was established which was associated with VAT1-related radioresistance in gliomas. High-risk patients showed higher proportion of elders, high-grade glioma, oligodendroglial histology and IDH wild type. The risk score was identified as an independent prognostic factor in the CGGA dataset, and the high-risk score impaired the overall survival time. The biological processes of positively expressed genes of risk score were functionally involved in inflammatory and immune response. And the activation of signaling pathways in high-risk score group also showed close correlation with tumor occurrence, progression and immune microenvironment. What's more, the immune cell infiltration analysis showed that high-risk score indicated decreased CD8^+^ T cell and the upregulation of the immune checkpoints, which probably promoted the immunosuppressive microenvironment.

**Conclusion:**

The five-gene signature can predict the survival of patients with glioma received postoperative radiotherapy efficiently. The immunosuppressive microenvironment, as a feature of glioma, potentially devote to the radioresistance.

## 1 Introduction

Glioma is a high malignancy primary intracranial tumor in adult with invasive fast-growing, high recurrence rate and fatality. The median survival of glioblastoma (GBM) was 14.6 months after comprehensively postoperative radiochemotherapy, and merely raised up to 20.9 months combining with the tumor-treating fields [1]. As a pivotal part of the treatment strategy, standard radiotherapy after resection is widely accepted, especially for high-grade tumors or low-grade tumors with risk factors. However, it was inevitable that patients would finally suffer from the relapse after radiotherapy, and more than 80 % of the relapse pattern occurred within the irradiation field [2], which limited the possibility of re-resection or re-administration of radiotherapy.

Radiotherapy is an efficient adjuvant strategy for gliomas that strengthens local control and prolongs survival. However, radioresistance is a characteristic potentially impacting patient survival that cannot be ignored. The mechanisms responsible for radioresistance in gliomas have yet to be elucidated, which may include stem cells, tumor microenvironment, hypoxia, DNA damage repair, and extracellular vesicles [3]. There were plenty of clinical trials evaluating different agents that capitalized on different pathways to enhance the efficacy of ionizing radiation. However, most substances either failed to improve patient outcome when compared to standard strategy or lacked validation via phase III trials with large cohorts. Thus, the radiotherapy with or without concomitant and adjuvant temozolomide (TMZ) remains the standard therapy for most gliomas [4].

Vesicle amino transport protein 1 (VAT1), localizing in cytoplasm and mitochondria, was identified as potential prognostic factor in patients with glioma. The gene oncology annotation labeled that VAT1 possessed oxidoreductase activity and regulated mitochondrial fusion. The positively related genes of VAT1 functionally participated in the immune response, oxidation-reduction process, and hypoxia response in our previous study [5]. Moreover, we previously found the positive correlation between VAT1 expression and some immune checkpoints and verified in vitro assays, indicating the immunosuppressive function of VAT1 in gliomas [6]. Taking the above-mentioned reasons, we screened out a VAT1-related radioresistant signature using the transcriptional dataset to comprehensively explore the interaction between VAT1 and radiotherapy in gliomas, and further excavated its clinical value.

## 2 Materials and methods

### 2.1 Data collection

A total of 325 patients from the Chinese Glioma Genome Atlas (CGGA, http://www.cgga.org.cn/) with complete clinicopathological and RNA sequencing data were included [7]. Seven hundred and two patients with clinical and transcriptional data were obtained from The Cancer Genome Atlas (TCGA, https://portal.gdc.cancer.gov). The inclusion criteria including age ≥18 years old, primary tumor, received postoperative radiotherapy and survival time ≥30 days. Finally, 191 patients with whole grade gliomas from the CGGA database were eligible for the training set, and 430 patients from the TCGA database were used for the validation.

### 2.2 Construction and the validation of gene signature

The median survival time was used as the cutoff to define the differentially expressed genes. The correlation analysis between VAT1 expression and positively expressed genes of patients with worse survival was subsequently conducted. Ultimately, 3707 VAT1 related radioresistant genes (r ≥ 0.3) were selected as the gene set for the least absolute shrinkage and selection operator (Lasso) regression analysis. The risk score was then calculated based on the regression coefficient (β) by applying the following formula: risk score = expression of gene 1∗ coefficient 1 + expression of gene 2 ∗ coefficient 2 + … + expression of gene n ∗ coefficient n. Afterwards, the patients with whole grade gliomas were dichotomized into high-risk and low-risk groups according to the median risk score.

### 2.3 Bioinformatics analysis

Gene Ontology (GO) analysis and Kyoto Encyclopedia of Genes and Genomes (KEGG) pathways analyses were conducted between the high-risk and low-risk groups with genes’ fold change ranging top 500 (https://david.ncifcrf.gov/tools.jsp).

### 2.4 GSEA and immune cells infiltration analysis

To further identify the activation of the related signaling pathways, we conducted the gene set enrichment analysis (GSEA), and the background gene symbols were downloaded from the public website (https://www.gsea-msigdb.org/gsea/msigdb/human/collections.jsp#C2). The gene symbol of different immune cells was obtained from a published article [8]. Gene set variation analysis (GSVA) then used so identify the immune cell infiltration status with the variation of risk score.

### 2.5 Validation of the protein expression level of radioresistant gene signature

The immunohistochemistry (IHC) data acquired from the Human Protein Atlas (HPA, https://www.Proteinatlas.org) was applied to demonstrate the protein expression level of the five signature genes.

### 2.6 Statistical analysis

R language (version 4.3.0, http://www.r-project.org) was applied to statistical analysis and graph generation (R packages including glmnet, ggplot2, survivalROC, GSVA, ComplexHeatmap, regplot, etc.). Comparation between two groups were conducted using the Wilcoxon rank sum and two-tailed Student's *t* tests. Chi-square test was performed to show the differences in clinicopathological characteristics between groups. Pearson correlation analysis was used to screen out the VAT1 related radioresistant genes. The prognostic value of the five-genes signature was exhibited via Kaplan-Meier (K-M) curve using GraphPad Prism software. The overall survival (OS) was defined as the time from surgery date to death of any causes. Patients lost to follow-up were censored at the time of last known contact. Statistical significance was considered when p < 0.05.

## 3 Results

### 3.1 Construction of the five-gene signature

**Fig. 1:**
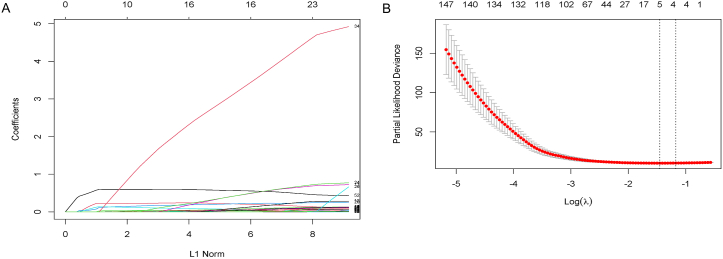
The construction of the VAT1-related radioresistant gene signature. (A) LASSO coefficient profiles of the common genes. (B) Cross-validation for tuning parameter screening in the LASSO regression model. VAT1, vesicle amino transport protein 1; LASSO, least absolute shrinkage and selection operator.

**Fig. 2:**
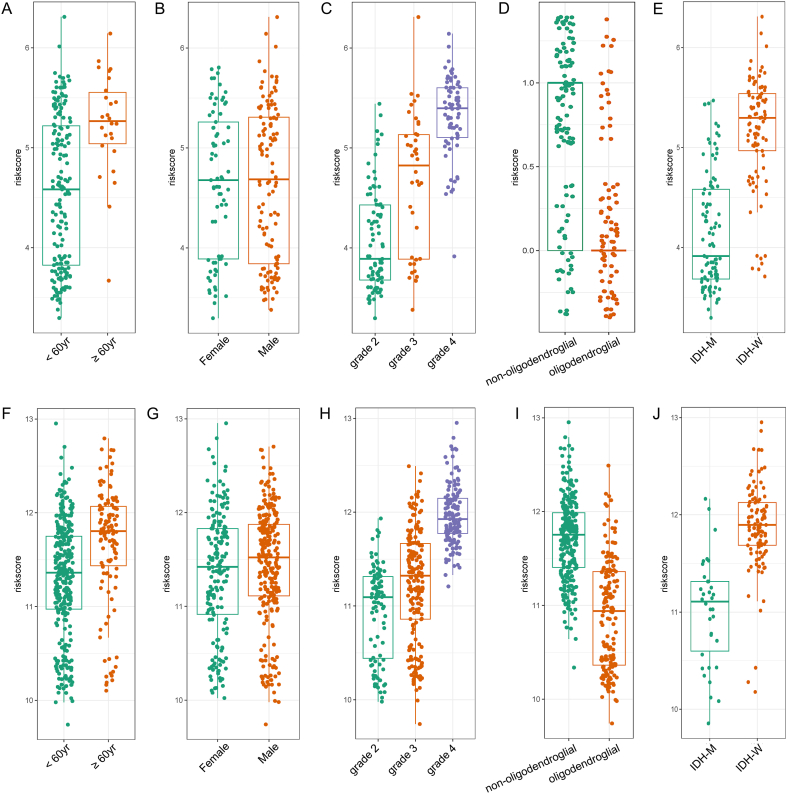
The clinicopathological features between high-risk score group and low-risk score group. (A, F) age; (B, G) gender; (C, H) WHO grade; (D, I) histology; (E, J) IDH status. (A–E) from CGGA database. (F–J) from TCGA database. WHO, the World Health Organization; IDH, isocitric dehydrogenase; CGGA, the Chinses Glioma Genome Atlas; TCGA, The Cancer Genome Atlas.

Total of 191 patients with whole grade glioma from the CGGA database were included in this study as the training set, and 430 patients from the TCGA database conformed to the inclusion criteria were used for validation. The whole patients received postoperative radiotherapy and were divided into two groups according to their OS time. The screening result was demonstrated in Fig. 1A and B that five genes were selected (*TXLNA, DDOST, GNG5, TXNDC12* and *ARAP3*), and the corresponding coefficients were used to calculate the risk score. The same equation was applied in both CGGA and TCGA database. The whole patients were divided into high-risk and low-risk groups based on the risk score. Patients in the high-risk group had more elders, high-grade glioma, oligodendroglial histology and isocitrate dehydrogenase (IDH) wild type (Table 1, Fig. 2A–J, Supplementary Table 1). Patients with O6-methylguanine-DNA methyltransferase (MGMT) promoter methylation received postoperative chemotherapy accounted for higher proportion in high-risk group.

**Table 1:** The clinicopathological characteristics between high-risk score group and low-risk score group in CGGA database.

Characteristics		High-risk score (n = 96)	Low-risk score (n = 95)	P value
Age (yr)	<60	73	92	**<0.001**
	≥60	23	3	
Gender	Male	59	59	0.927
	Female	37	36	
Gross total resection	Yes	50	49	0.098
	No	28	46	
WHO grade	2	11	70	**<0.001**
	3	21	19	
	4	64	6	
Histology	Oligodendroglial	19	63	**<0.001**
	Non-oligodendroglial	77	32	
IDH mutation	Yes	19	80	**<0.001**
	No	77	15	
1p/19q codeletion	Yes	0	48	**<0.001**
	No	95	46	
MGMT promoter methylation	Yes	33	44	**0.001**
	No	50	22	
Chemotherapy	Yes	66	40	**0.001**
	No	30	50

### 3.2 Prognostic value of the five-gene signature

**Fig. 3:**
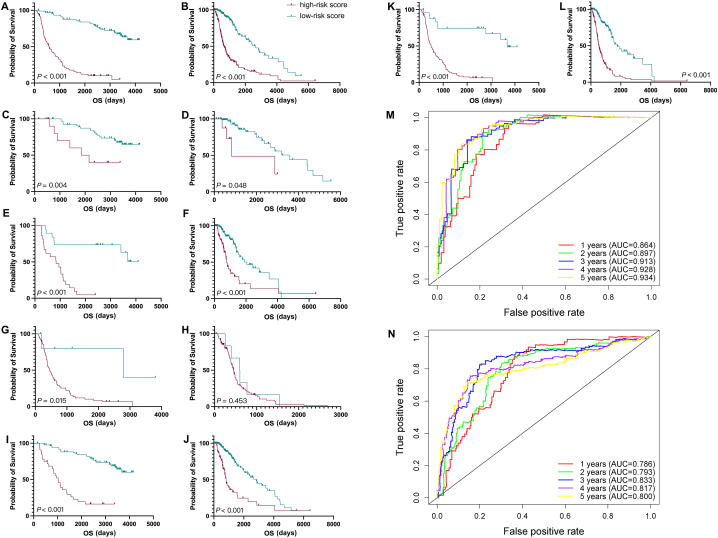
The prognostic value of risk score in OS. The K-M curves of high-risk score and low-risk score group in the whole grade (A, B), grade 2 (C, D), grade 3 (E, F), grade 4 (G, H), lower-grade (I, J) and high grade (K, L) gliomas. (A, C, E, G, I, K) from CGGA database. (B, D, F, H, J. L) from TCGA database. The time-dependent ROC analysis of the sensitivity and specificity of the survival for the five-genes signature in CGGA (M) and TCGA (N). OS, overall survival; K-M, Kaplan-Meier; CGGA, the Chinses Glioma Genome Atlas; TCGA, The Cancer Genome Atlas; ROC, receiver operating characteristic.

The Kaplan-Meier survival curve showed that patients with low-risk score had better prognosis than high-risk group in the whole grade group, and the similar results were verified in different grades (Fig. 3A–L). While the survival differences between high- and low-risk groups in patients with grade 4 from the TCGA database did not have statistical significance (Fig. 3H). Time-dependent receiver operating characteristic (ROC) curves revealed the one-to five-year OS. The whole AUC values in the training group were over 0.864, of which the five-year area under curve (AUC) value reached up to 0.934 (Fig. 3M). In the TCGA validation group, risk score also demonstrated a better prediction of one-to five-year survival (Fig. 3N).

### 3.3 Bioinformatic analysis

**Fig. 4:**
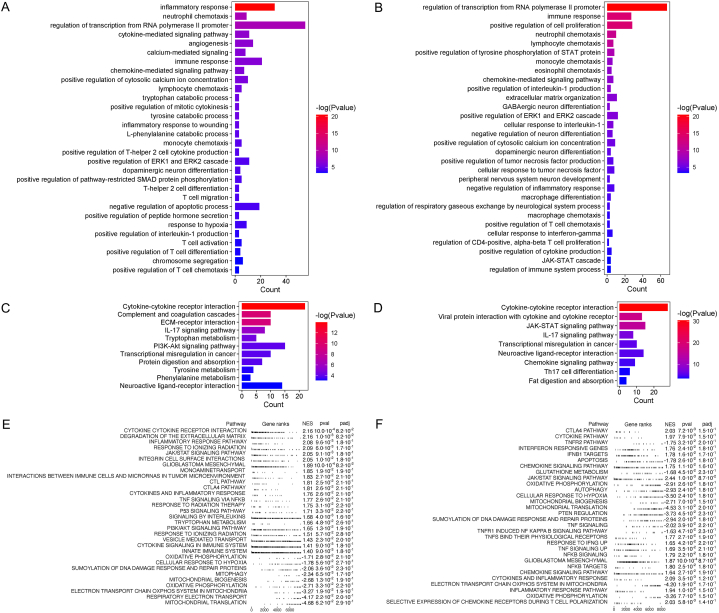
The function annotation of differentially expressed genes in high-risk score. The functionally involved biological processes (A, B) and pathways (C, D) from GO analysis. The activated pathways from GSEA analysis (E, F). (A, C, E) from CGGA database. (B, D, F) form TCGA database. GO, gene oncology; GSEA, gene set enrichment analysis; CGGA, the Chinses Glioma Genome Atlas; TCGA, The Cancer Genome Atlas.

To further explore the roles of the genes positively correlated with the risk score, we conducted a series of gene function and pathway enrichment analyses. The result of GO analysis indicated the close correlation with inflammatory and immune response, cytokine- and chemokine-mediated signaling pathways as well as certain amino acid metabolism pathways (Fig. 4A and B). The result of KEGG analysis revealed that the cytokine-cytokine receptor interaction was the most correlated pathway in both CGGA and TCGA database (Fig. 4C and D). Besides, IL-17 signaling pathway, transcriptional misregulation in cancer and neuroactive ligand-receptor interaction were also the common pathways in both databases. The GSEA analysis results showed the signaling pathways activating condition more comprehensively, which involved in immune checkpoint pathway, immune cells pathway, response to radiotherapy, response to hypoxia, oxidative phosphorylation and mitochondrial-related pathways, etc. (Fig. 4E and F).

### 3.4 Univariate and multivariate analysis

**Fig. 5:**
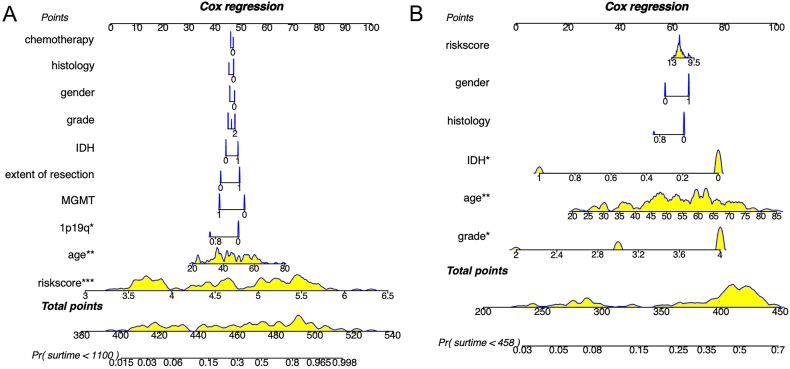
Nomograms based on the result of Cox regression analysis in CGGA (A) and TCGA (B). CGGA, the Chinses Glioma Genome Atlas; TCGA, The Cancer Genome Atlas. ∗p < .05, ∗∗p < .01, ∗∗∗p < .001.

In the subsequent univariate and multivariate Cox regression analysis, we assessed the prognostic value of clinicopathological characteristics. The nomogram showed that the risk score (p < 0.001), age and 1p/19q were independent prognostic factors in the CGGA dataset (Fig. 5A). In the TCGA dataset, risk score was statistically significant in univariate analysis, while only age and tumor grade were still significant after multivariate analysis (Fig. 5B).

### 3.5 Immune cell infiltration

**Fig. 6:**
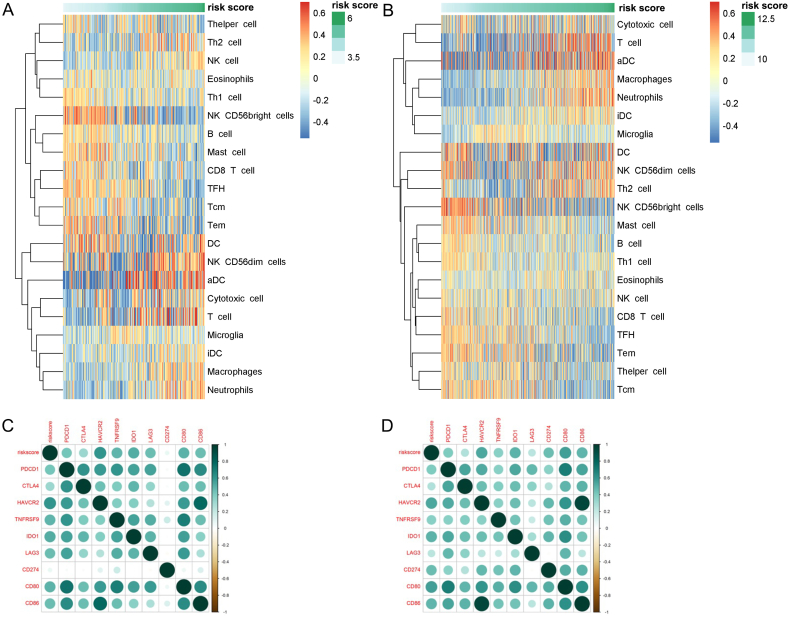
The infiltration of immune cells (A, B) and the immune checkpoint expression (C, D) based on the risk score. (A, C) from CGGA database. (B, D) form TCGA database. CGGA, the Chinses Glioma Genome Atlas; TCGA, The Cancer Genome Atlas.

The immune cell infiltration in tumor microenvironment was analyzed by GSVA. Significant increases were identified in immune cell infiltration of dendritic cells (DCs), T cells, macrophages and neutrophils in the high-risk score group, which may indicate the more activate immune microenvironment (Fig. 6A and B). Conversely, immune cells exerting anti-tumor activity were decreased in the high-risk score group, such as CD8^+^ T cells. The correlation analysis result demonstrated that the risk score was positively correlated to the expression of immune checkpoints, including *PDCD1*, *CTLA4*, *TIM-3*, etc. (Fig. 6C and D).

### 3.6 The function and protein expression of the five genes

**Fig. 7:**
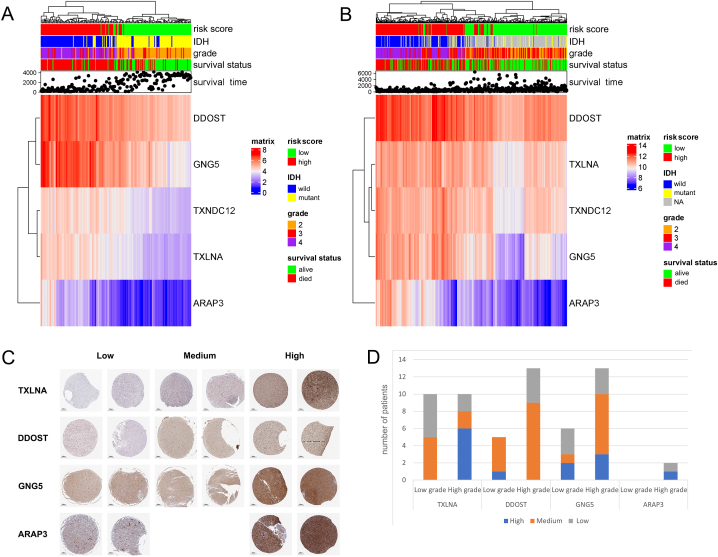
The heatmap of the five genes expression corresponding to the risk score, IDH, tumor grade and survival status in CGGA (A) and TCGA (B). Protein expression of the five genes in HPA database (C) and the number of patients based on the gene expression and tumor grade (D). Scale bars = 200 μm. IDH, isocitric dehydrogenase; CGGA, the Chinses Glioma Genome Atlas; TCGA, The Cancer Genome Atlas; HPA, Human Protein Atlas.

The annotation of the five genes indicated that the function was mainly involved in mediating protein binding and exerting enzyme activity (Supplementary Table 2). The clustering results showed the expression of five genes vary with the risk score, tumor grade and IDH status (Fig. 7A and B). The immunohistochemical results downloaded from the HPA database were used to exhibit the protein expression level of four genes among different patients (Fig. 7C and D). The protein information corresponding to TXNDC12 was unavailable on the website. The protein expression level was classified as low, medium and high, and the bar chart indicating larger proportion of high protein expression level of signature genes in patients with high grade glioma (WHO grade 3 and 4 mentioned in the HPA database). In accordance with the RNA sequencing data, higher grade meant higher risk score.

## 4 Discussion

Glioma is a high malignancy tumor with strong aggressiveness and inevitable recurrence. In addition to surgery and systemic chemotherapy, radiotherapy is essential treatment method for gliomas. Radiation plays an important role in attacking tumor cells via some well-established pathways. After ionizing radiation, tumor cells could be killed directly by inducing DNA double strand breaks (DSBs), and indirectly by promoting immunogenic cell death (ICD) [9]. The initiation of inflammatory chemokines secretion and the recruitment of immune cells then activated the immune response, and ultimately led to the tumor cell death. Meanwhile, tumor antigens released by necrotic cells could also stimulate the immune response [10]. However, the immune response generated by radiation could be beneficial or adverse in that the radiation could stimulate both anti-tumor immune response and the immunosuppressive response. Plenty of reports revealed that the recruitment of tumor-associated macrophages (TAMs), myeloid-derived suppressor cell (MDSC) and regulatory T cell (Treg) could cause the suppression of T-cell immunity and impede the efficacy of radiotherapy [11,12]. Meanwhile, immune checkpoints could be upregulated after radiation, which prevented the stimulation of anti-tumor immune response and accelerated the T cell exhaustion. To counteract this problem, many clinical trials focused on the combination treatment of radiotherapy and immune checkpoints inhibitors. Nevertheless, the complex immune microenvironment and the low expression of immune checkpoints resulted in the failure in large-scale of clinical trials for GBMs [13,14].

Additionally, the repair of DNA damage, hypoxia and cancer stem cells (CSCs) were also generally recognized as major obstacles to radiotherapeutic efficacy. Several radiosensitizers aim to stabilize DNA damage by preventing or interfering cellular repair mechanisms. The poly ADP-ribose polymerase (PARP) proteins are intracellular mediators exerting discovery and management of DNA damage by activating pathways of homologous recombination and nonhomologous end-joining [15,16]. Several studies have evaluated the effect of PARP inhibitor in gliomas suggesting its potential radiosensitizing as well as chemosensitizing properties [4]. Hypoxia as a hallmark of glioma plays an important role in radioresistance, diminishing the damage caused by ironizing radiation. Hypoxia microenvironment could induce the epithelial-to-mesenchymal transition (EMT) promoting tumor cells’ resistance to apoptotic stimuli, which subsequently resulted in radioresistance [17]. Moreover, the cancer stem cells (CSCs) also exerted radioresistant role in glioma. It has been illustrated that CSCs could enrich both in vitro and in vivo after ironizing radiation, indicating their innately higher radioresistant capacity [18].

Previously, we identified that the high expression of VAT1, as a prognostic marker of gliomas, promoted the generation of tumor immunosuppressive microenvironment. Considering the influence of radiation on immune response in gliomas, we established a VAT1 related five-gene signature in patients receiving radiotherapy. The K-M survival analysis showed the effectiveness of the five genes impacting OS. The ROC curves demonstrated that the risk score could robustly predict one-to five-year OS. Multivariate Cox regression analysis identified the five-gene signature as an independent prognostic factor in CGGA dataset. The immune response seemed more activated in high-risk score group that the immune cells infiltration was positively correlated with the risk score. Moreover, the expression of immune checkpoints also upregulated with the increase of risk score, which indicated strongly immunosuppressive effect in the high-risk score group.

The five genes have been investigated respectively that they played key roles in tumor progression and immunosuppression. Taxilin alpha (TXLNA), also known as interleukin 14 (IL-14), highly expressed in various tumor cells, is a binding partner of the syntaxin family that can coordinate intracellular vesicle trafficking [14]. High TXLNA expression predicted unfavorable outcome for GBMs [19]. Encoding a component of the oligosaccharide transferase complex, Dolichyl-diphosphooligosaccharide protein glycosyltransferase non-catalytic subunit (DDOST) was related to the N‐glycosylation of proteins and mediated the immunosuppressive microenvironment of gliomas [20]. G protein subunit gamma 5 (GNG5), a subunit of G-protein, promoting the proliferation and migration of tumor cells, was also related to the immune activity [21]. Thioredoxin domain protein 12 (TXNDC12) encodes a protein which is functionally involved in catalyzing the formation of disulfide bonds and played an important role in endoplasmic reticulum stress. TXNDC12 was highly expressed in gliomas and positively associated with immune cell infiltration, playing a vital role in tumor occurrence and progression [22]. ARAP3, highly expressed in neutrophils, is a GTPase activating protein for the small GTPases RhoA and Arf6. As an effector of PI3K, ARAP3 regulated the integrin inactivation and further affected the recruitment and function of neutrophils [23]. In combination of the above, the individual study has detailed gene's function and its potential impact on gliomas. Therefore, patients with high-risk score received an earlier and higher dose radiotherapy, or the combination of radiotherapy and other strategies, might be appropriate.

Nevertheless, despite the application of bioinformatics analysis and large databases, the study has some limitations. Firstly, the public database was retrospective in nature, and the samples decreased after screening the radiotherapy information. Secondly, lots of complex biological processes involved in radioresistance, which could be investigated combining with the 5-gene signature in the future.

## 5 Conclusions

This research established a five-gene signature that could predict the survival of patients with glioma received postoperative radiotherapy efficiently. The immunosuppressive microenvironment, as a feature of glioma, potentially devote to the radioresistance.

## CRediT authorship contribution statement

**Xia Shan:** Writing – original draft, Formal analysis, Data curation. **Zhiyan Sun:** Software, Methodology, Data curation. **Ruoyu Huang:** Software, Methodology, Data curation. **Kuanyu Wang:** Writing – review & editing, Software, Methodology, Data curation. **Xiaoguang Qiu:** Writing – review & editing, Supervision, Conceptualization. **Pei Yang:** Writing – review & editing, Supervision, Funding acquisition, Conceptualization.

## Data availability statement

The data that support the findings of this study are available from the corresponding author upon reasonable request.

## Ethical declaration

CGGA, TCGA and HPA belong to public databases. The patients involved in the database have obtained ethical approval. Users can download relevant data for free for research and publish relevant articles. Our study is based on publicly available data, so there are no ethical issues and other conflicts of interest.

## Funding

10.13039/501100012427Beijing Tiantan Hospital Miaopu Project (2023MP09 to X.S.), Beijing Municipal Administration of Hospitals Incubating Program (PX2024019 to P.Y.).

## Declaration of competing interest

The authors declare the following financial interests/personal relationships which may be considered as potential competing interests: Pei Yang reports financial support was provided by 10.13039/501100009601Beijing Municipal Administration of Hospitals Incubating Program. Xia Shan reports financial support was provided by Beijing Tiantan Hospital Miaopu Project. If there are other authors, they declare that they have no known competing financial interests or personal relationships that could have appeared to influence the work reported in this paper.
