# Supplementary material for: A VAT1-related gene signature predicts radioresistance in gliomas
Source: Heliyon. 2025 Feb 8;11(4):e42583. doi: 10.1016/j.heliyon.2025.e42583 (PMC11875813; doi:10.1016/j.heliyon.2025.e42583)
Supplement: Multimedia component 1 [file mmc1.docx]

**Supplementary table 1. The clinicopathological characteristics between high-risk score group and low-risk score group in TCGA database**

| Characteristics | | High-risk score  (n=215) | Low-risk score  (n=215) | P value |
| --- | --- | --- | --- | --- |
| Age (yr) | < 60 | 130 | 184 | **<0.001** |
|  | ≥ 60 | 85 | 31 |  |
| Gender | Male | 131 | 121 | 0.328 |
|  | Female | 84 | 94 |  |
| WHO grade | 2 | 8 | 84 | **<0.001** |
|  | 3 | 76 | 125 |  |
|  | 4 | 131 | 6 |  |
| Histology | Oligodendroglial | 29 | 135 | **<0.001** |
|  | Non-oligodendroglial | 186 | 80 |  |
| IDH mutation | Yes | 10 | 33 | **<0.001** |
|  | No | 143 | 15 |  |

**Supplementary table 2. Genes function of the five genes downloaded from the website (https://www.genecards.org/)**

| **Gene** | **Function** | **GO ID** | **GO term** |
| --- | --- | --- | --- |
| TXLNA | May be involved in intracellular vesicle traffic and potentially in calcium-dependent exocytosis in neuroendocrine cells. | GO：0005515 | enables protein binding |
|  |  | GO：0019905 | enables syntaxin binding |
| DDOST | Subunit of the oligosaccharyl transferase (OST) complex that catalyzes the initial transfer of a defined glycan (Glc(3)Man(9)GlcNAc(2) in eukaryotes) from the lipid carrier dolichol-pyrophosphate to an asparagine residue within an Asn-X-Ser/Thr consensus motif in nascent polypeptide chains, the first step in protein N-glycosylation (PubMed:31831667). N-glycosylation occurs cotranslationally and the complex associates with the Sec61 complex at the channel-forming translocon complex that mediates protein translocation across the endoplasmic reticulum (ER). All subunits are required for a maximal enzyme activity (By similarity). Required for the assembly of both SST3A- and SS3B-containing OST complexes (PubMed:22467853). | GO：0005515 | enables protein binding |
|  |  | GO：0008047 | enables enzyme activator activity |
| GNG5 | Guanine nucleotide-binding proteins (G proteins) are involved as a modulator or transducer in various transmembrane signaling systems. The beta and gamma chains are required for the GTPase activity, for replacement of GDP by GTP, and for G protein-effector interaction. | GO：0003924 | enables GTPase activity |
|  |  | GO：0005515 | enables protein binding |
|  |  | GO：0030165 | enables PDZ domin binding |
|  |  | GO：0031681 | enables G-protein beta-subunit binding |
| TXNDC12 | Protein-disulfide reductase of the endoplasmic reticulum that promotes disulfide bond formation in client proteins through its thiol-disulfide oxidase activity. | GO：0005515 | enables protein binding |
|  |  | GO：0015035 | enables protein-disulfide reductase activity |
|  |  | GO：0016491 | oxidoreductase activity |
|  |  | GO：0019153 | enables protein-disulfide reductase (glutathione) activity |
| ARAP3 | Phosphatidylinositol 3,4,5-trisphosphate-dependent GTPase-activating protein that modulates actin cytoskeleton remodeling by regulating ARF and RHO family members. Is activated by phosphatidylinositol 3,4,5-trisphosphate (PtdIns(3,4,5)P3) binding. Can be activated by phosphatidylinositol 3,4-bisphosphate (PtdIns(3,4,5)P2) binding, albeit with lower efficiency. Acts on ARF6, RAC1, RHOA and CDC42. Plays a role in the internalization of anthrax toxin. | GO：0005096 | not enables GTPase activator activity |
|  |  | GO：0005515 | enables protein binding |
|  |  | GO：0005547 | enables phosphatidylinositol-3,4,5-trisphosphate binding |
|  |  | GO：0043325 | enables phosphatidylinositol-3,4-bisphosphate binding |
|  |  | GO：0046872 | enables metal ion binding |
